# Understanding the effects of *Haemophilus influenzae* colonization on bronchiectasis: a retrospective cohort study

**DOI:** 10.1186/s12890-023-02823-8

**Published:** 2024-01-02

**Authors:** Seo-Hee Yang, Myung Jin Song, Yeon Wook Kim, Byoung Soo Kwon, Sung Yoon Lim, Yeon-Joo Lee, Jong Sun Park, Young-Jae Cho, Jae Ho Lee, Choon-Taek Lee, Hyung-Jun Kim

**Affiliations:** 1 Division of Pulmonary and Critical Care Medicine, Department of Internal Medicine, Seoul National University Bundang Hospital, 82, Gumi-ro 173 Beon-gil, Bundang-gu, Seongnam, Gyeonggi-do 13620 Republic of Korea; 2 Department of Internal Medicine, Seoul National University College of Medicine, 103, Daehak-ro, Jongno-gu, Seoul, 03080 Republic of Korea; 3 Division of Pulmonary and Critical Care Medicine, Department of Internal Medicine, Seoul Veterans Hospital, 53, Jinhwangdo-ro 61-gil, Gangdong-gu, Seoul, 05368 Republic of Korea

**Keywords:** Bronchiectasis, Haemophilus influenzae, Pseudomonas, Propensity scores, Prognostic factor

## Abstract

**Background:**

Bacterial colonization is an essential aspect of bronchiectasis. Although *Haemophilus influenzae* is a frequent colonizer in some regions, its clinical impacts are poorly understood. This study aimed to elucidate the impact of *H. influenzae* colonization in patients with bronchiectasis.

**Methods:**

This retrospective study screened adult patients diagnosed with bronchiectasis at a tertiary referral center between April 1, 2003, and May 16, 2021, in South Korea. Propensity score matching was used to match patients with and without *H. influenzae* colonization. We assessed the severity of bronchiectasis as per the bronchiectasis severity index, the incidence of exacerbation, differences in lung function, and all-cause mortality.

**Results:**

Out of the 4,500 patients with bronchiectasis, 79 (1.8%) were colonized by *H. influenzae*. After 1:2 propensity score matching, 78 and 154 patients were selected from the *H. influenzae* colonizer and non-colonizer groups, respectively. Although there were no significant differences between the groups regarding baseline demographics, patients colonized with *H. influenzae* had a higher bronchiectasis severity index (median 6 [interquartile range 4–8] vs. 4 [2–7], *p* = 0.002), associated with extensive radiographic involvement (52.2% vs. 37.2%, *p* = 0.045) and mild exacerbation without hospitalization (adjusted incidence rate ratio 0.15; 95% confidence interval 0.12–0.24). Lung function and mortality rates did not reveal significant differences, regardless of *H. influenzae* colonization.

**Conclusion:**

*H. influenzae* colonization in bronchiectasis was associated with more severe disease and greater incidence of mild exacerbation, but not lung function and mortality. Attention should be paid to patients with bronchiectasis with *H. influenzae* colonization.

**Supplementary Information:**

The online version contains supplementary material available at 10.1186/s12890-023-02823-8.

## Background

Bronchiectasis is characterized by structural abnormalities of the airways, accompanied by bronchial tissue destruction [1]. Symptoms of bronchiectasis include recurrent respiratory infections that require antibiotics, cough with phlegm, shortness of breath, and intermittent hemoptysis [2]. Chronic inflammation and infection of the bronchi and airways lead to chronic bacterial colonization, which further causes inflammation and structural damage to the airways, creating the so-called “vicious cycle” of bronchiectasis [3]. There are only a few evidence-based treatments for bronchiectasis, and they are all primarily aimed at treating bacterial colonization [4].

The predominant colonizers in bronchiectasis are *Pseudomonas aeruginosa* and *Haemophilus influenzae* [5]. The microbiome shows regional variation: *P. aeruginosa* is the most prevalent species in the U.S., whereas, in Europe, it is *H. influenzae* [6]. Both bacteria can form biofilms that protect the bacteria from the body’s immune system or reduce systemic delivery of antibiotics, increasing antibiotic resistance and causing an inflammatory response, leading to additional airway damage, thereby creating a vicious cycle [1, 7].

It is well known that *P. aeruginosa* colonization contributes to pulmonary dysfunction and frequent exacerbations and is associated with high mortality risk [8]. *H. influenzae* was repeatedly cultured in stable chronic obstructive pulmonary disease (COPD) patients with bronchiectasis [9]. Chronic *H. influenzae* colonizers show increased inflammation and disease activity [10]. Considering that both bronchiectasis and COPD are respiratory diseases of a chronic nature, we hypothesized that *H. influenzae* colonization could be of clinical importance.

Despite the high prevalence of *H. influenzae* in bronchiectasis in some regions, the clinical impact of this colonization is not well known [5]. We aimed to evaluate the effects of *H. influenzae* colonization on the characteristics and clinical outcomes of patients diagnosed with bronchiectasis.

## Methods

### Study design and patient selection

This was a single-center, retrospective cohort study of patients with bronchiectasis at Seoul National University Bundang Hospital, Republic of Korea. Adult (> 18 years) patients were screened according to their International Classification of Diseases-10 codes in the electronic health records system. We reviewed medical records and selected patients with a diagnosis of bronchiectasis who had chest computed tomography (CT) results and sputum cultures. To rule out the influence of nontuberculous mycobacteria, the importance of which has already been well established, patients in whom these bacteria were identified were excluded. Patients included in this study were selected from those who submitted sputum for further testing. The method of sputum collection involved patients expectorating sputum spontaneously, without adherence to a pre-defined protocol. The day of bronchiectasis diagnosis was defined as the date on which the diagnosis of bronchiectasis was entered into the electronic medical record system, and the date of the most recent outpatient visit was determined as the date of the last follow-up. Patients were divided into two groups according to the presence of *H. influenzae* from sputum culture during their stable status [11].

### Propensity score matching and data collection

Due to the largely imbalanced data, we used a propensity score-based method to reduce the effects of disturbances in observational studies. The propensity score matching process was performed using the following variables to ensure comparability between groups: age, sex, colonization of *P. aeruginosa* or other bacteria, follow-up duration, and the number of pulmonary function tests [12, 13].

After matching, demographics, comorbidities, radiographic, laboratory, and microbiological findings were reviewed, and the bronchiectasis severity index (BSI) was calculated [14]. The BSI consists of age, body mass index (BMI), forced expiratory volume in 1 s (FEV_1_), hospitalization history, exacerbation frequency, degree of breathlessness assessed by the modified Medical Research Council dyspnea scale, bacterial colonization status, and radiographic findings [14]. Comorbidities included chronic lung diseases such as COPD and asthma and systemic diseases possibly associated with bronchiectasis such as rheumatoid arthritis and inflammatory bowel disease. The cause of bronchiectasis was defined as infectious if there was a past or childhood infection such as tuberculosis, pertussis, or measles, and idiopathic if there was no specific history [5]. Bacterial colonization was defined as the detection of bacteria at least once from the sputum sample during a stable status [11].

The Seoul National University Bundang Hospital Institutional Review Board approved this study (protocol number B-2106-689-106) and waived the need for informed consent owing to the study’s observational nature and the use of anonymized data. This study was conducted in accordance with the Declaration of Helsinki.

### Outcome measures

Exacerbation of bronchiectasis was defined as acute aggravation of respiratory symptoms such as changes in sputum nature, shortness of breath, increased cough or fatigue, and hemoptysis. Mild exacerbation was defined as that requiring an outpatient prescription of oral antibiotics, and severe exacerbation was defined as a worsening course and hospitalization or embolization [14]. Incidence rate was used to effectively report the frequency of acute exacerbations in patients with bronchiectasis, and negative binomial regression analysis was performed to compare exacerbation incidence rates between groups.

Pulmonary function indicators, including FEV_1_, forced vital capacity (FVC), and FEV_1_/FVC ratio, and the results obtained at an outpatient clinic within two years before and after diagnosis were adopted as the baseline pulmonary function. The date of death was obtained from data requested by the Ministry of Public Administration and Security. Linear mixed regression analyses were performed to compare the repeated measures of pulmonary function.

### Other statistical considerations

A standard (unconditional) analysis was performed and considered valid [15]. Simple descriptive statistics of the mean with standard deviation were used for continuous parametric data, median with interquartile range (IQR) was used for continuous nonparametric data, and frequencies and percentages for categorical data. Subgroup comparisons were performed using the chi-squared test, Fisher’s exact test, and Mann-Whitney U Test, depending on data distribution. Kaplan-Meier curves and log-rank tests were used for survival analyses [16]. The Division of Statistics in the Medical Research Collaborating Centre reviewed and approved the statistical analyses at Seoul National University Bundang Hospital. The REporting of studies Conducted using Observational Routinely-collected Data checklist is available in the online supplement [see Additional file 1].

## Results

### Propensity score matching and patient characteristics

**Fig. 1:**
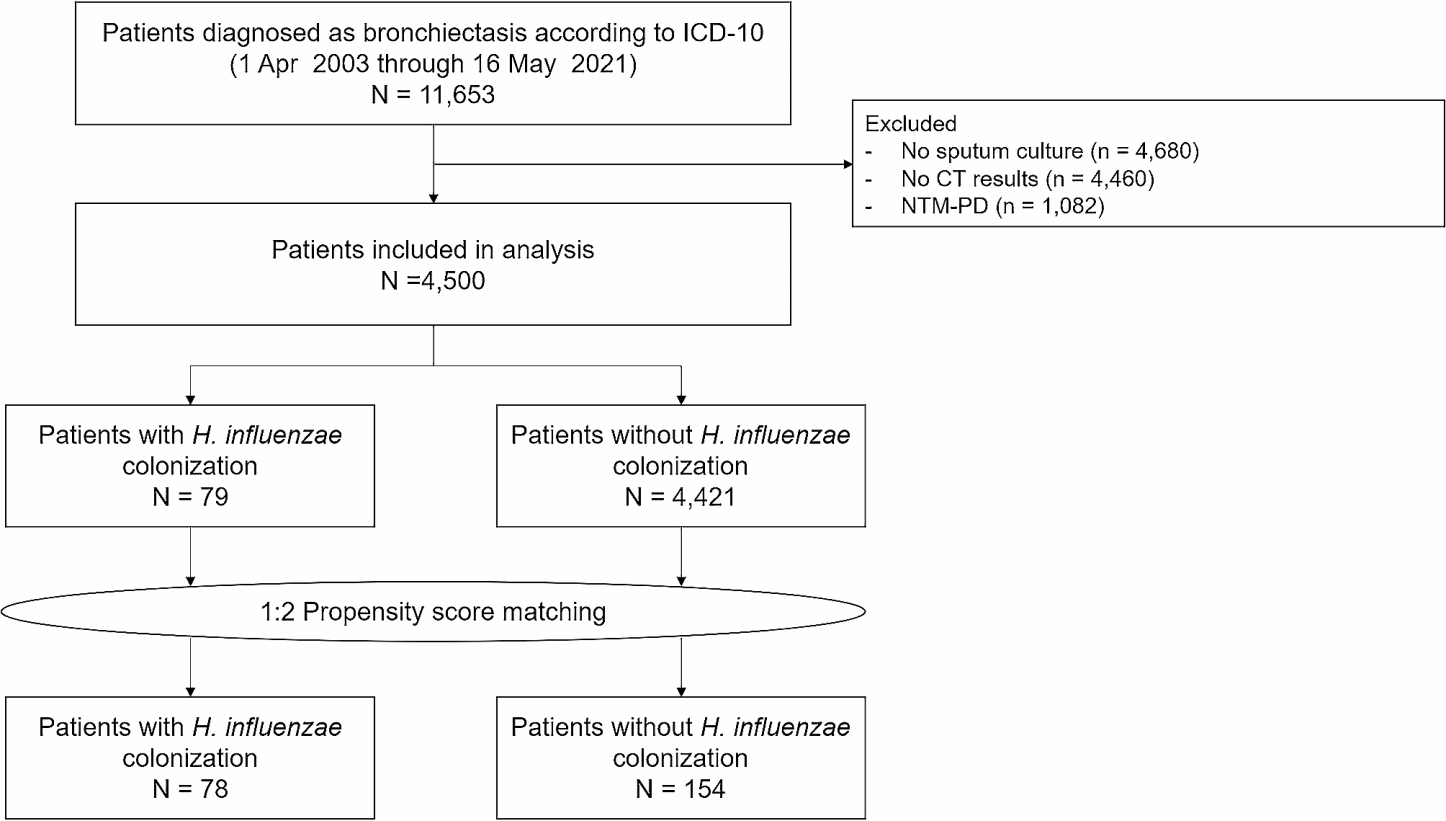
Flowchart of the patient selection process

Among the patients who visited the outpatient department of pulmonology from April 1, 2003, to May 16, 2021, 11,653 patients were screened to be diagnosed with bronchiectasis according to the International Classification of Diseases-10 in our electronic health records system. After excluding 4,680 patients without sputum culture results, 4,460 patients without CT scans, and 1,082 patients with nontuberculous mycobacteria, 4,500 adult patients were included in the analysis (Fig. 1).

Out of the 4,500 patients, 79 (1.8%) were colonized by *H. influenzae*. After 2:1 propensity score matching of the remaining 4,421 patients, 156 were selected. *H. influenzae* colonizer (*n* = 78) and non-colonizer (*n* = 156) groups showed significant differences in terms of *P. aeruginosa* colonization (*p* < 0.001), colonization with organisms other than *H. influenzae and P. aeruginosa* (*p* = 0.001), duration of follow-up (*p* < 0.001), and the number of pulmonary function tests performed (*p* = 0.005). The significant differences observed between the two groups are listed in Table 1.

**Table 1:** Patient characteristics before and after matching

Variables	Before matching			After matching		
	*H. Influenzae* colonizers	*H. Influenzae* non-colonizers	*P*	*H. Influenzae* colonizers	*H. Influenzae* non-colonizers	*P*
	n = 79	n = 4,421		n = 78	n = 154	
Age	59.0 [51.5–65.0]	60.0 [52.0–67.0]	0.298	59.0 [52.0–65.0]	58.5 [51.0–66.0]	0.977
Sex, male	32 (40.5)	1859 (42.0)	0.873	32 (41.0)	64 (41.6)	> 0.999
Colonization of *Pseudomonas aeruginosa*	24 (30.4)	549 (12.4)	< 0.001	23 (29.5)	45 (29.2)	> 0.999
Colonization of any other bacteria	37 (46.8)	1293 (29.2)	0.001	36 (46.2)	65 (42.2)	0.665
Follow-up duration, years	7.90 [2.56–13.39]	4.02 [1.14–8.40]	< 0.001	7.73 [2.52–13.37]	9.10 [2.68–13.90]	0.919
Number of PFTs, counts	2.00 [1.00–5.00]	1.00 [1.00–3.00]	0.050	2.00 [1.00–5.00]	2.00 [1.00–5.00]	0.486

Data are shown as count (percentage) or median [interquartile range]. Abbreviations: PFT, pulmonary function test

In the overall patient group, the median age was 59 (IQR, 51–66) years, and there was a female predominance (58.6%). The most common comorbidities were a history of tuberculosis (34.4%), hypertension (21.6%), and COPD (19.1%). There were no significant differences between the two groups regarding BMI (*p* = 0.233), smoking history (*p* = 0.497), underlying comorbidities, or possible causes of bronchiectasis. The patient characteristics of the two groups are described in detail in Table 2.

**Table 2:** Baseline characteristics of bronchiectasis patients selected after propensity score matching

Variables	Total	*H. Influenzae* colonizers	*H. Influenzae* non-colonizers	*P*
	N = 232	n = 78	n = 154	
**Age, years**	59.00 [51.00–66.00]	58.50 [51.00–66.25]	59.00 [51.75–65.00]	0.977
**Male sex**	96 (41.4)	32 (41.0)	64 (41.6)	> 0.999
**BMI, kg/m** ^**2**^	21.55 [19.80–25.29]	22.41 [19.41–25.26]	22.65 [20.63–25.40]	0.233
**Smoking**				0.497
Never smoker	179 (74.3)	60 (76.9)	119 (77.3)	
Former smoker	25 (10.4)	6 (7.7)	19 (12.3)	
Current smoker	27 (11.2)	12 (15.4)	15 (9.7)	
**Comorbidities**				
TB history	83 (34.4)	25 (32.1)	58 (37.7)	0.382
Hypertension	52 (21.6)	18 (23.1)	34 (22.1)	0.884
COPD	46 (19.1)	21 (26.9)	25 (16.2)	0.074
Asthma	38 (15.8)	18 (23.1)	20 (13.0)	0.073
**Possible cause of bronchiectasis**				
Idiopathic	142 (58.9)	49 (62.8)	93 (60.4)	0.765
Infectious	88 (36.5)	29 (37.2)	59 (37.7)	0.769

Data are shown as count (percentage) or median [interquartile range]. Abbreviations: BMI, Body Mass Index; TB, Tuberculosis; COPD, Chronic obstructive pulmonary disease

### Comparison of BSI score

The total BSI score was significantly higher in the *H. influenzae* colonizer group (median 6 [IQR, 4–8] vs. 4 [IQR, 2–7], *p* = 0.002). Patients colonized by *H. Influenzae* had more frequent exacerbations (40.6% vs. 18.6%, *p* = 0.002) and more extensive radiographic involvement (52.2% vs. 37.2%, *p* = 0.045). However, age (*p =* 0.676), BMI (*p =* 0.898), FEV_1_ (*p* = 0.204), modified Medical Research Council dyspnea scale (*p* = 0.321), and *P. aeuruginosa* colonization rate (*p = 0.725*) were not significantly different between the two groups (Table 3).

**Table 3:** Comparison of bronchiectasis severity index according to the colonization of *Haemophilus influenzae*

Variables	Score points	Total	*H. Influenzae* colonizers	*H. Influenzae* non-colonizers	*P*
		N = 198	n = 69	n = 129	
Age, years					0.676
<50	0	42 (21.2)	14 (20.3)	28 (21.7)	
50–69	2	129 (65.2)	46 (66.7)	83 (64.3)	
70–79	4	21 (10.6)	9 (13.0)	12 (9.3)	
80+	6	6 (3.0)	0 (0.0)	6 (4.7)	
BMI < 18.5 kg/m^2^	2	25(12.6)	16 (12.4)	9 (13.0)	0.898
FEV_1_, % predicted					0.204
>80	0	108 (54.5)	34 (49.3)	74 (57.4)	
50–80	1	70 (35.4)	27 (39.1)	43 (33.3)	
30–49	2	18 (9.1)	6 (8.7)	12 (9.3)	
<30	3	2 (1.0)	2 (2.9)	0 (0.0)	
Hospital admission before study within two years	5	16 (8.1)	7 (10.1)	9 (7.0)	0.438
Exacerbations before study within two years ≥ 3	2	52 (26.3)	28 (40.6)	24 (18.6)	0.002
MRC dyspnea score					0.321
0	0	197 (99.5)	68 (98.6)	129(100.0)	
1–2	2	1 (0.5)	1 (1.4)	0 (0.0)	
≥3	3	0 (0.0)	0 (0.0)	0 (0.0)	
Pseudomonas colonization	3	60 (30.3)	22 (31.9)	38 (29.5)	0.725
Colonization with other organisms	1	137 (69.2)	69 (100.0)	68 (52.7)	< 0.001
≥ 3 lobes involved or cystic bronchiectasis	1	84 (42.4)	36 (52.2)	48 (37.2)	0.045
Total BSI score	26	5 [3–8]	6 [4–8]	4 [2–7]	0.002

Data are shown as count (percentile) or median [interquartile range] unless specified otherwise. Abbreviations: BMI, Body Mass Index; FEV1, forced expiratory volume in one second; mMRC, modified Medical Research Council dyspnea scale; BSI, bronchiectasis severity index

### Risk of exacerbations

**Fig. 2:**
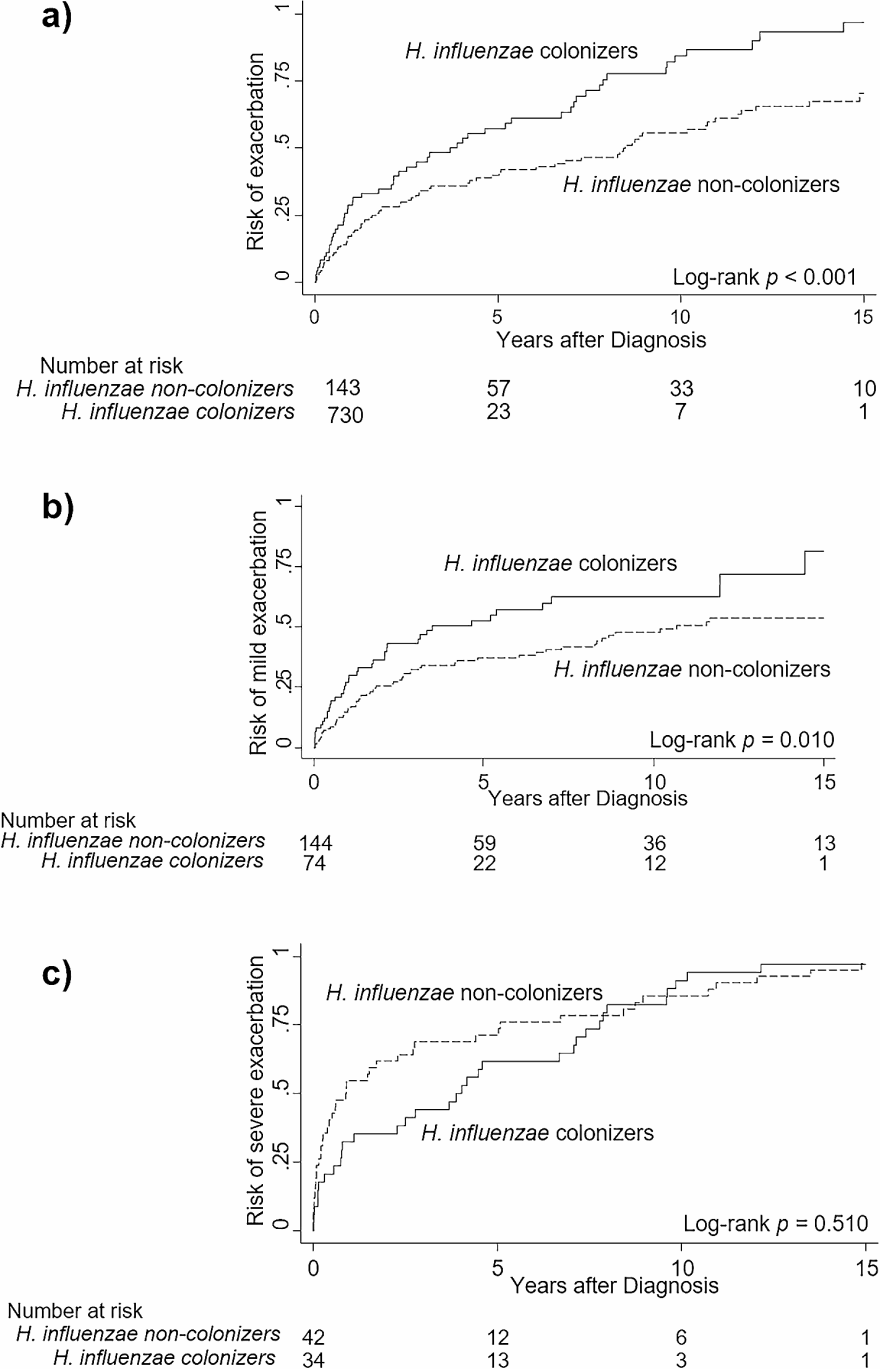
Effect of colonization of *Haemophilus influenzae* on the risk of exacerbation of bronchiectasis. The lines indicate Kaplan-Meier curves assessing the risk of exacerbation after diagnosis. Solid lines indicate patients with *Haemophilus influenzae* colonization, while dashed lines indicate patients without colonization. Compared to patients without colonization, patients with *Haemophilus influenzae* colonization had higher risk of (**a**) any exacerbation, defined as worsening of respiratory symptoms such as cough, increased sputum, or requiring antibiotics or hospitalization (*p* < 0.001) and (**b**) mild exacerbation requiring prescription of oral antibiotics from an outpatient clinic (*p* = 0.010). However, no difference was observed regarding (**c**) severe exacerbation requiring hospitalization or emergency room visits (*p* = 0.510)

Patients with *H. influenzae* colonization had a shorter time to overall exacerbation than those without *H. influenzae* colonization (log-rank test, *p* < 0.001); when exacerbation was split into two categories, the difference was maintained for mild exacerbation (log-rank test, *p* = 0.010) but not for severe exacerbation. (Log-rank test, *p* = 0.510) (Fig. 2). After multivariate adjustment, *H. influenzae* colonization (incidence rate ratio [IRR] 1.54, 95% confidence interval [CI] 1.06–2.24, *p* = 0.023), *P. aeruginosa* colonization (adjusted IRR 1.70, 95% CI 1.12–2.56, *p* = 0.012), underlying asthma (adjusted IRR 1.77, 95% CI 1.10–2.85, *p* = 0.019), and involvement of more than three lobes (adjusted IRR 1.72, 95% CI 1.14–2.57, *p* = 0.009) were revealed to have a significant association with the incidence rate of mild exacerbation (Table 4).

**Table 4:** Factors associated with the incidence rate of mild exacerbation^*^ in bronchiectasis patients

Variables	Unadjusted IRR	*P*	Adjusted IRR	*P*
*H. influenzae* colonization^†^	1.63 [1.08–2.44]	0.019	1.54 [1.06–2.24]	0.023
*P. aeruginosa* colonization^†^	2.04 [1.38–3.03]	< 0.001	1.70 [1.12–2.56]	0.012
Bacteria other than *H. influenzae* and *P. aeruginosa* colonization	1.90 [1.27–2.84]	0.002	1.31 [0.86–1.99]	0.208
BMI (kg/m^2^)	0.93 [0.88–0.99]	0.023	0.96 [0.91–1.02]	0.190
Chronic obstructive pulmonary disease	1.85 [1.18–2.91]	0.008	1.03 [0.67–1.58]	0.896
Asthma	1.71 [1.02–2.86]	0.041	1.77 [1.10–2.85]	0.019
Hospitalization history within two years	2.25 [1.22–4.14]	0.009	1.52 [0.86–2.66]	0.148
Involvement ≥ 3 lobes	2.50 [1.69–3.68]	< 0.001	1.72 [1.14–2.57]	0.009

^*^Mild exacerbation refers to an outpatient prescription of oral antibiotics due to exacerbation. ^†^Colonization was defined as when any type of bacteria was detected at least once from respiratory specimen culture during routine practice. Data are shown as median [interquartile range] unless specified otherwise. Abbreviations: IRR, incidence rate ratio; BMI, Body Mass Index

Abbreviations: ICD, International Classification of Diseases; CT, computed tomography; NTM-PD, Non-tuberculous mycobacterial pulmonary disease

### Comparison of lung function and overall survival

**Fig. 3:**
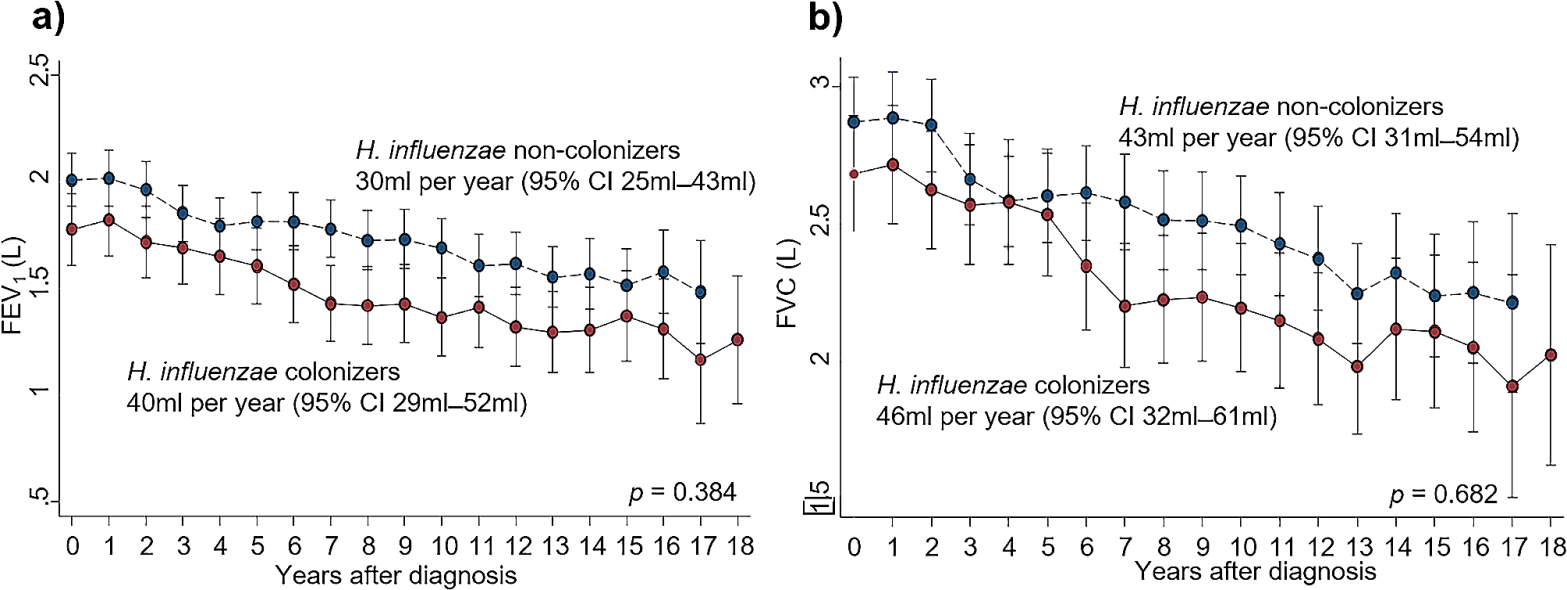
Decline of pulmonary function according to the colonization of *Haemophilus influenzae* in bronchiectasis patients. The annual decline of (**a**) FEV_1_ (*p* = 0.382) and (**b**) FVC (*p* = 0.628) did not show significant differences regardless of *Haemophilus influenzae* colonization in bronchiectasis patients. Solid lines indicate patients with *Haemophilus influenzae* colonization, while dashed lines indicate patients without colonization. Abbreviations: FEV1, forced expiratory volume in one second; FVC, forced vital capacity; CI, confidence interval

The baseline and annual changes in FEV_1_ and FVC revealed similar findings regardless of *H. influenzae* colonization status. The baseline FEV_1_ was 1.70 L (IQR 1.25–2.30 L) vs. 1.98 L (IQR 1.50–2.44) (*p* = 0.082), and FVC was 2.65 L (IQR 2.06–3.27 L) vs. 2.81 L (IQR 2.30–3.33 L) (*p* = 0.170) for *H. influenzae* colonizers and non-colonizers, respectively. The annual decline in FEV_1_ was 40 ml (95% CI 29–52 ml) for *H. influenzae* colonizers, while it was 30 ml (95% CI 25–43 ml) for non-colonizers, which was not a statistically significant difference (*p* = 0.384). The decline in FVC was 46 ml (95% CI 32–61 ml) per year for *H. influenzae* colonizers and 43 ml (95% CI 31–54 ml) per year for non-colonizers (*p* = 0.682) (Fig. 3).

**Fig. 4:**
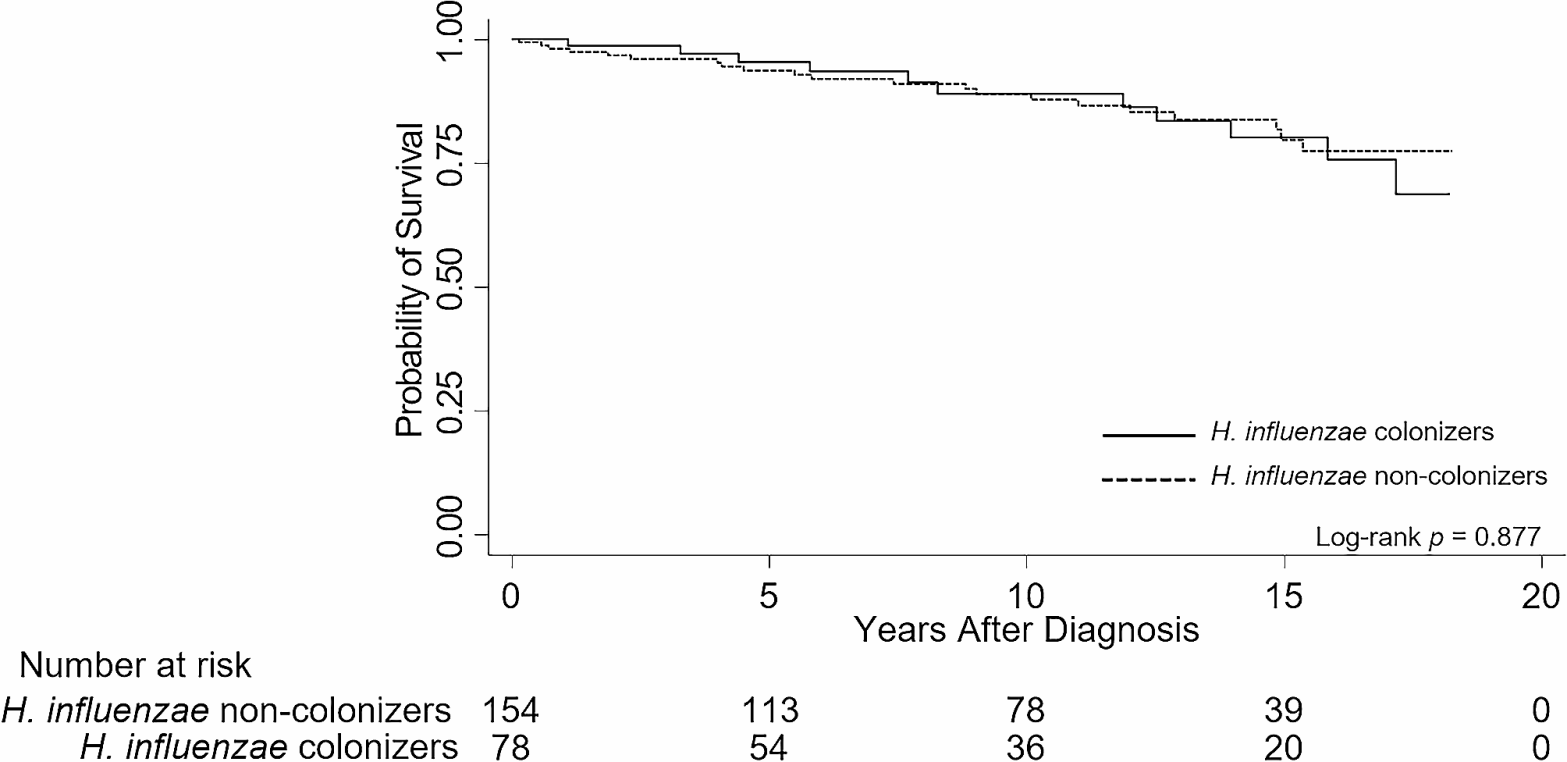
Impact of *Haemophilus influenzae* colonization on the all-cause mortality of bronchiectasis patients. Colonization of *Haemophilus influenzae* did not have a significant impact on all-cause mortality of bronchiectasis patients (log-rank *p* = 0.877). Solid lines indicate patients with *Haemophilus influenzae* colonization, while dashed lines indicate patients without colonization

The probability of survival did not differ according to the colonization status of *H. influenzae* (log-rank test, *p* = 0.877). The Kaplan-Meier survival curve is shown in Fig. 4.

## Discussion

This study evaluated the impact of *H. influenzae* colonization on the clinical features and prognosis of patients with bronchiectasis. Patients with bronchiectasis colonized by *H. influenzae* had higher BSI scores, attributed to more extensive radiological involvement and a higher frequency of mild exacerbations. Lung function and mortality rates did not differ significantly according to the presence of *H. influenzae.*

This is the first study to evaluate the impact of *H. influenzae* colonization on bronchiectasis. According to our results, *H. influenzae* colonization did not affect lung function or mortality; however, there was a difference in the frequency of exacerbations. In particular, a higher incidence of mild exacerbation in the *H. influenzae* colonization group implies a lower quality of life and frequent hospital visits, although hospitalization was not required. The grave impact of *H. influenzae* colonization can be inferred from previous studies on patients with COPD. COPD patients colonized with *H. influenzae* had increased airway inflammation and decreased lung volume compared to non-colonizers [11]. Also, it is known to be common in patients with moderate-to-severe COPD [17]. Taken together, *H. influenzae* colonization in patients with bronchiectasis is likely to induce more airway inflammatory responses and contribute to a poor prognosis. Although the effects of other respiratory diseases or coinfection were not studied in depth in this study, we have tried to outline the significant impact of *H. influenzae* colonization. In addition, considering that *H. influenzae* is often not cultured during disease exacerbation [9], this study indicates the importance of a culture study to acquire colonization information, especially that of *H. influenzae*, in the initial treatment of patients with bronchiectasis.

In this study, *H. influenzae* colonization did not affect severe exacerbation, overall mortality, or changes in lung function. In individuals with intact immunity, *H. influenzae* usually causes upper respiratory tract diseases. Lower respiratory tract infections are rare because of host immune responses that prevent transmission to the lower respiratory tract. When the mucosal host immune mechanism is compromised, lower respiratory tract infections can occur via the immune evasion mechanism of *H. influenzae* [18]. Indeed, the high rates of *H. influenza*e infection in conditions such as cystic fibrosis or immotile ciliary syndrome, characterized by abnormal mucociliary function, further emphasize the importance of these host defense mechanisms [19, 20]. In our study, most patients were followed up at the outpatient clinic, implying normal immunity rather than an immune-compromised situation. Therefore, the probability of identifying *H. influenzae* may have been lower, and there may not have been a significant difference owing to the small number of patients. However, identifying *H. influenzae* may indicate an abnormal immune system inside the bronchi, making it necessary to pay more attention while devising a treatment plan.

A common feature of *P. aeruginosa* and *H. influenzae* is their ability to form biofilms, facilitating antibiotic resistance, but they are not equally virulent. This may be due to the wide range of virulence factors and proinflammatory properties of *P. aeruginosa* [21]. In addition, *H. influenzae* is a common resident of the upper respiratory tract, which may mean that its presence is less destructive to the lower respiratory tract environment [22].

Despite these meaningful findings, our study has several limitations. First, this was a retrospective study. Regular follow-ups and detailed evaluations regarding the etiology of bronchiectasis could not be performed. Second, patients who did not undergo sputum culture were excluded, leading to a possible selection bias and limited generalizability. Third, the detection rate of *H. influenzae* was lower than expected. Based on previous studies, the most commonly observed bacterial pathogens in patients with bronchiectasis were *Haemophilus spp.* (19–55%) *Pseudomonas spp*. (26–58%) and *Streptococcus pneumoniae* (12%) [7, 23]. This may be due to the difficult culture process of *H. influenzae*. Usually, a standard method using chocolate agar medium is used for *H. influenzae* culture [24], which has high sensitivity, but a low specificity due to possible contamination with other bacterial species. Our study adopted a selective culture method using a medium supplemented with vancomycin, bacitracin, and clindamycin. Therefore, the specificity is higher, but the culture sensitivity may be lower [25]. Finally, our study did not consider the use of inhaled corticosteroids in patients with bronchiectasis. Future research efforts may benefit from incorporating inhaled corticosteroids as a covariate to further elucidate its specific impact on bronchiectasis severity and exacerbation risk.

## Conclusions

In conclusion, colonization of *H. influenzae* in bronchiectasis was associated with a higher risk of mild exacerbation but not severe exacerbation, lung function decline, or all-cause mortality. Although further evaluations are necessary, colonization with *H. influenzae* may have a harmful impact on patients with bronchiectasis.

### Electronic supplementary material

Below is the link to the electronic supplementary material.

Supplementary Material 1

## Data Availability

The datasets used and/or analyzed during the current study are available from the corresponding author on reasonable request.

## Abbreviations

COPD: Chronic obstructive pulmonary disease
BSI: Bronchiectasis severity index
BMI: Body mass index
FEV1: Forced expiratory volume in 1 s
IRR: Incidence rate ratio
FVC: Forced vital capacity
IQR: Interquartile range
CI: Confidence interval
