# Correction: Understanding the effects of Haemophilus influenzae colonization on bronchiectasis: a retrospective cohort study

**DOI:** 10.1186/s12890-024-02881-6

**Published:** 2024-02-13

**Authors:** Seo-Hee Yang, Myung Jin Song, Yeon Wook Kim, Byoung Soo Kwon, Sung Yoon Lim, Yeon-Joo Lee, Jong Sun Park, Young-Jae Cho, Jae Ho Lee, Choon-Taek Lee, Hyung-Jun Kim

**Affiliations:** 1 Division of Pulmonary and Critical Care Medicine, Department of Internal Medicine, Seoul National University Bundang Hospital, 82, Gumi-ro 173 Beon-gil, Bundang-gu, 13620 Seongnam, Gyeonggi-do Republic of Korea; 2 Department of Internal Medicine, Seoul National University College of Medicine, 103, Daehak-ro, Jongno-gu, 03080 Seoul, Republic of Korea; 3 Division of Pulmonary and Critical Care Medicine, Department of Internal Medicine, Seoul Veterans Hospital, 53, Jinhwangdo-ro 61-gil, 05368 Gangdong-gu, Seoul, Republic of Korea

**BMC Pulmonary Medicine (2023) 24:7**

10.1186/s12890-023-02823-8

Following publication of the original article [1], the authors flagged the following two concerns:

Affiliation 3 should read as follows:

Division of Pulmonary and Critical Care Medicine, Department of Internal Medicine, Seoul Veterans Hospital, 53, Jinhwangdo-ro 61-gil, Gangdong-gu, Seoul, 05368, Republic of Korea.

The total number of patients should be 4,500, not 4,453.

The published article has since been updated to correct for these errors. The authors thank you for reading and apologize for any inconvenience caused.

